# Influence of patient-related factors and surgeon experience on functional outcomes in conservatively treated proximal humerus fractures: a retrospective cohort study

**DOI:** 10.1007/s00590-026-04920-w

**Published:** 2026-08-25

**Authors:** Andrea Pautasso, Chiara Bernardi, Marco Assom, Davide Blonna, Federica Romano, Raffaele Garofalo, Alessandro Marinelli, Paolo Paladini, Filippo Castoldi, Enrico Bellato, SICSeG (Italian Society of Shoulder and Elbow Surgery) Research Committee

**Affiliations:** 1 ASST Sette Laghi, Orthopaedic and Traumatology Department, Ospedale di Circolo e Fondazione Macchi, Varese, Italy; 2 University of Insubria, Varese, Italy; 3 Orthopaedic and Traumatology Department, A. O. Ordine Mauriziano di Torino, Turin, Italy; 4 CIR Dental School, Department of Surgical Sciences, University of Turin, Turin, Italy; 5 Orthopaedic and Trauma Surgery Department, IRCCS Humanitas Research Hospital, Rozzano, Italy; 6 Shoulder and Sport Medicine Unit, Ospedale Generale Regionale Francesco Miulli, Acquaviva delle Fonti, Italy; 7 Shoulder and Elbow Unit, Istituto Ortopedico Rizzoli, Bologna, Italy; 8 Shoulder and Elbow Unit, Ospedale Cervesi di Cattolica, Cattolica, Italy; 9 Department of Surgical Sciences, University of Turin, Turin, Italy; 10 Orthopaedic and Traumatology Department, Ospedale San Luigi Gonzaga, Orbassano, Italy; 11 Orthopaedic and Traumatology Department, Ospedale di Ciriè-Lanzo, Turin, Italy

**Keywords:** Proximal humerus fractures, Conservative treatment, Shoulder surgeon, Traumatology

## Abstract

**Purpose:**

Proximal humerus fractures (PHFs) are common in the elderly population, but the optimal treatment strategy remains controversial. This study evaluated the long-term functional outcomes of non-surgically treated PHFs and assessed whether retrospective treatment recommendations made by experienced shoulder surgeons were associated with differences in clinical outcomes.

**Materials and methods:**

This retrospective cohort study included 150 PHFs in 149 patients treated nonoperatively between 2020 and 2021. Two-, three-, and four-part fractures with a minimum 12-month follow-up were analyzed. Outcome measures included QuickDASH, Oxford Shoulder Score (OSS), Subjective Shoulder Value (SSV), and SF-12. Demographic characteristics and radiographic fracture features were analyzed. In a second phase, five shoulder surgeons independently reviewed anonymized cases and retrospectively recommended surgical or conservative treatment while blinded to patient outcomes.

**Results:**

At a mean follow-up of 32.1 months, younger age, absence of comorbidities, better pre-injury physical status, and dominant limb involvement were independently associated with superior outcomes, whereas the number of fracture fragments did not. Displaced fractures showed worse outcomes than non-displaced fractures. However, among displaced fractures, no significant differences in SSV, OSS, QuickDASH, or SF-12 scores were found between fractures retrospectively considered surgical candidates and those deemed suitable for conservative management.

**Conclusions:**

Functional recovery following conservative treatment of PHFs appears to be influenced more by patient-related factors than by fracture morphology or surgeon experience. These findings suggest that carefully selected patients with displaced PHFs can achieve satisfactory outcomes with nonoperative treatment.

**Levels of evidence:**

IV.

## Introduction

Proximal humeral fractures (PHFs) are among the most common fractures in the elderly population (aged over 65 years [1]). They account for approximately 5% of all fractures and represent the third most common osteoporotic fracture after hip and distal radius fractures. Their incidence is steadily increasing due to the progressive aging of the population [2–4].

The majority of PHFs are managed nonoperatively, with conservative treatment reported in up to 80% of cases in large epidemiological and clinical studies. Despite their high prevalence and the extensive body of literature on their management, the optimal treatment strategy remains a matter of debate [5].

Several factors contribute to this lack of consensus. First, no currently available classification system for PHFs has demonstrated both high prognostic or therapeutic value and satisfactory inter-observer reproducibility [6, 7]. This limitation has been consistently documented in the literature, as both the Neer and AO/OTA classification systems show variable and often moderate reproducibility, which hinders their reliability for clinical decision-making and comparative research [6–8].

Secondly, surgical management encompasses a broad spectrum of procedures, ranging from minimally invasive fixation techniques to shoulder arthroplasty. Furthermore, substantial technical variability exists even within the same procedure. For example, in reverse shoulder arthroplasty, the decision to repair the tuberosities remains controversial and may influence functional outcomes [9].

Finally, the publication of the ProFHER study has further contributed to uncertainty; despite its known methodological limitations, including patient selection and heterogeneity of surgical techniques, its findings have supported a more conservative treatment approach, particularly among orthopedic surgeons not specializing in shoulder surgery, resulting in broader indications for non-surgical treatment [10].

Given these uncertainties, the extent to which surgeon experience and clinical judgment influence treatment recommendations and patient outcomes remains unclear. Therefore, the primary aim of this study was to evaluate the long-term functional outcomes of a cohort of patients with PHFs treated nonoperatively. A secondary aim was to determine whether the retrospective treatment recommendations of experienced shoulder surgeons identified a subgroup of patients who achieved different long-term functional outcomes compared with those considered appropriate for conservative management.

## Materials and methods

### Study design

A retrospective observational cohort study was conducted on patients with PHFs who underwent non-operative treatment. Eligible cases were identified among patients presenting to the Emergency Departments at AOU San Luigi Gonzaga Hospital, Orbassano (Italy).

All PHFs diagnosed and classified between January 1st, 2020, and December 31st, 2021, were screened for eligibility. Patients were included if they received a non-surgical treatment indication in the emergency department from on-call orthopedic surgeons, none of whom had specific sub-specialty training in shoulder surgery. Treatment decisions were made according to routine clinical practice taking into account multiple factors, including patient age, comorbidities, pre-injury functional demand, fracture morphology, degree of displacement, bone quality, and the surgeon's clinical judgement.

Patient demographic characteristics, injury-related data (date and side of injury), comorbidities, fracture characteristics, type of imaging obtained were extracted from the institutional database. Radiographic assessment included anteroposterior, Grashey, and transthoracic lateral views. Fractures were classified according to the number of fragments, the presence or absence of fracture displacement, and the presence or absence of humeral head splitting.

All patients were managed according to the institutional nonoperative treatment protocol, consisting of immobilization in a shoulder brace for four weeks, worn continuously day and night, followed by a standardized rehabilitation program.

Inclusion criteria were PHFs (two-, three-, or four-part fractures); initial non-operative treatment indication in the emergency department; completion of conservative treatment followed by rehabilitation and a minimum clinical follow-up of 12 months.

Exclusion criteria were isolated greater tuberosity fractures; diaphyseal fractures; fracture sequelae; subsequent surgical treatment; severe psychiatric or cognitive disorders precluding reliable clinical assessment, and incomplete radiographic documentation.

This first phase of the study was designed to evaluate the clinical and functional outcomes of the non-surgical approach.

In the second phase, five orthopedic surgeons specialized in upper limb surgery (MA, DB, RG, AM, PP) independently reviewed the anonymized radiological images and clinical information of all included fractures while blinded to clinical outcomes. Reviewers first classified each fracture as displaced or non-displaced according to their clinical judgment. Subsequently, fractures considered displaced were independently assessed to determine whether surgical or conservative treatment would have been recommended.

### Follow-up and statistical analysis

Functional and health-related quality of life outcomes were assessed using validated Italian versions of the following instruments: Quick Disabilities of the Arm, Shoulder and Hand (QuickDASH) [11]; Oxford Shoulder Score (OSS) [12]; Subjective Shoulder Value (SSV) [13]; Short Form-12 Health Survey (SF-12), including the Physical (PCS) and Mental (MCS) component summary [14].

Statistical analyses were performed using Jamovi software (Version 2.6; The jamovi Project, 2025). Continuous variables were tested for normality using the Shapiro–Wilk test. As the outcome variables were not normally distributed, continuous data are presented as median and interquartile range (IQR), while categorical variables are reported as frequencies and percentages. Accordingly, nonparametric statistical methods were used for all univariate analyses.

Comparisons between two independent groups, including gender (male vs. female), presence of comorbidities (yes vs. no), involvement of the dominant extremity (yes vs. no), and fracture displacement (displaced vs. non-displaced), were performed using the Mann–Whitney U test. Associations between continuous variables were assessed using Spearman's rank correlation coefficient.

To identify variables independently associated with functional outcomes, separate multiple linear regression models were constructed for each dependent variable. Independent variables entered into the models included gender, comorbidities, dominance of the injured extremity, fracture displacement, pre-injury physical status and age at injury. Regression coefficients (β), 95% confidence intervals (CIs), and *p* values were estimated using bias-corrected and accelerated (BCa) bootstrap resampling with 2000 iterations to obtain robust estimates. All statistical tests were two-sided, and statistical significance was defined as *p* < 0.05.

## Results

### Study Population

**Fig. 1:**
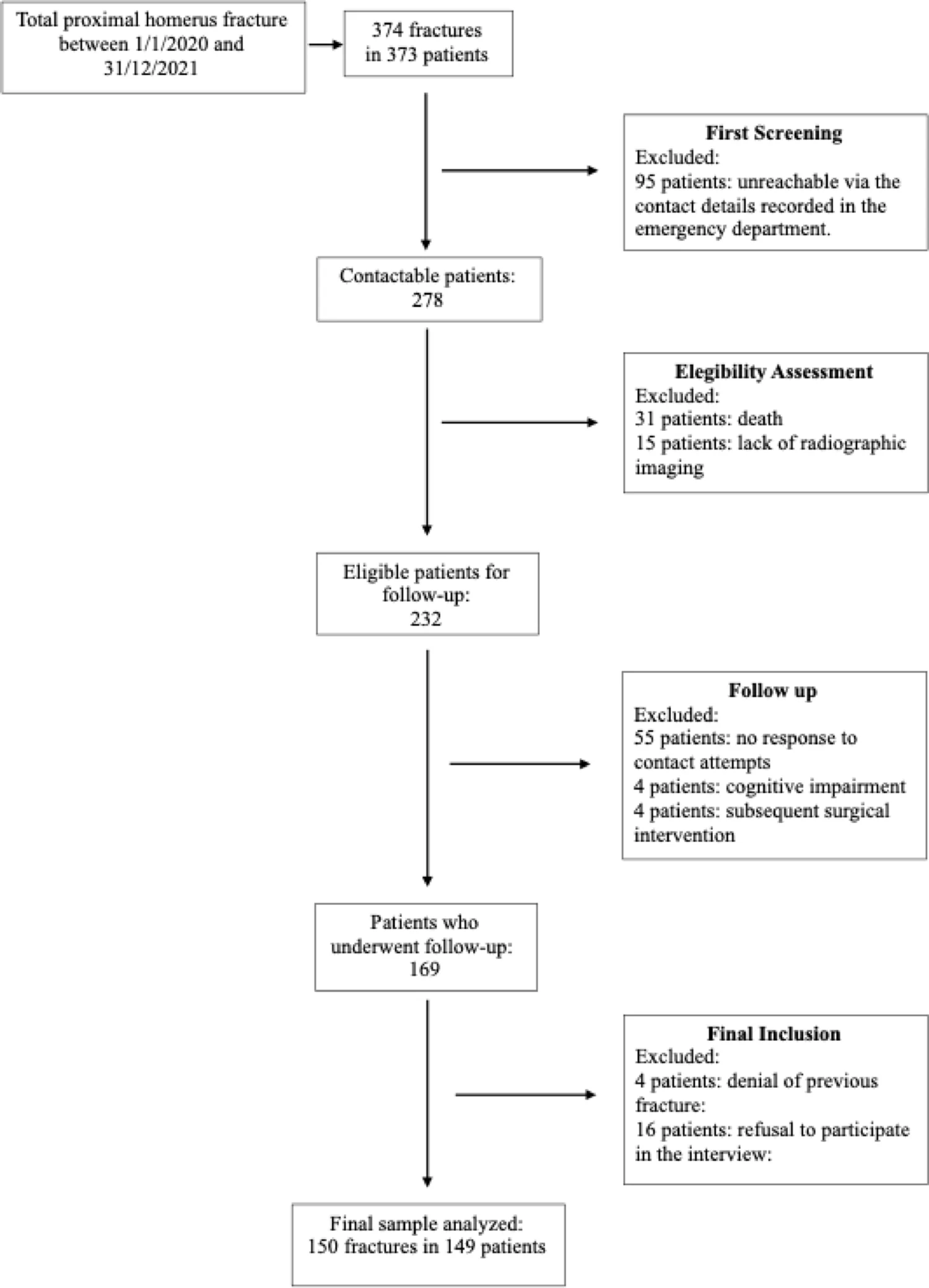
CONSORT flow-diagram for patients included in the study

We initially identified a total of 374 fractures in 373 patients. The final study cohort included 149 patients with 150 PHFs, as shown in Fig. 1.

Females comprised the majority of the cohort, accounting for 119 fractures (79.3%). The dominant limb was involved in 73 fractures (48.7%).

The mean age at the time of injury was 72.2 ± 11.5 years (range 25–90 years), while the mean age at follow-up was 74.9 ± 11.5 years (range 27–93 years). The average follow-up duration was 32.1 ± 6.7 months (range 22–49 months).

Forty-four patients (29.5%) had no documented comorbidities, whereas 105 (70.5%) had one or more comorbid conditions. The most common comorbidities were arterial hypertension (33.3%), cardiovascular disease (14.0%), malignancy (12.7%), and diabetes mellitus (11.3%).

### Functional outcomes

Patient-reported functional outcomes are summarized in Table 1.

**Table 1:** Functional outcomes in the overall study cohort

	*n* = 150
SSV	
Median (IQR)	85.0 (25.0)
Range	0–100
OSS	
Median (IQR)	41.0 (14.0)
Range	3–48
Quick dash	
Median (IQR)	11.4 (27.9)
Range	0–77.5
PCS post-trauma	
Median (IQR)	48.4 (21.7)
Range	19–63.8
MCS post trauma	
Median (IQR)	57.7 (10.5)
Range	20.3–66.5

Patient outcomes were afterwards analyzed according to demographic, clinical, and radiographic characteristics, as well as pre- and post-injury physical and mental health.

Female patients generally exhibited poorer functional scores than male patients, with statistically significant differences in OSS (*p* = 0.010) and QuickDASH (*p* = 0.044) scores (Table 2).

**Table 2:** Follow-up outcome measures stratified by gender (Mann–Whitney U test)

	Group	*N*	Median	IQR	*P*-value
SSV	Female	119	80.0	25.0	**0.101**
	Male	31	90.0	18.0	
OSS	Female	119	40.0	13.0	*0.010*
	Male	31	46.0	10.0	
Quick dash	Female	119	12.5	29.9	*0.044*
	Male	31	6.8	25.0	
PCS post-trauma	Female	119	48.0	21.3	**0.218**
	Male	31	52.1	23.9	
MCS post-trauma	Female	119	57.9	9.4	**0.978**
	Male	31	57.0	16.4	

Fractures involving the dominant limb were associated with better functional outcomes, with significantly higher SSV, OSS, and SF-12 PCS scores and lower QuickDASH than fractures involving the non-dominant limb (Table 3). Comorbidity status also influenced recovery. Patients without comorbidities demonstrated significantly better OSS, QuickDASH, and SF-12 PCS scores than those with at least one comorbidity (Table 4). Among individual comorbidities, only cardiovascular disease was associated with significantly lower SF-12 PCS scores, whereas hypertension, diabetes, and a history of cancer were not associated with significant differences in shoulder-specific outcomes.

**Table 3:** Follow-up outcome measures stratified by dominant limb (Mann–Whitney U Test)

	Group	*N*	Median	IQR	*P*-value
SSV	Not dominant limb	77	80.0	22.5	*0.028*
	Dominant limb	73	90.0	29.0	
OSS	Not dominant limb	77	39.0	15.0	*0.012*
	Dominant limb	73	44.0	11.0	
Quick Dash	Not dominant limb	77	15.0	35.2	*0.031*
	Dominant limb	73	7.5	22.7	
PCS post-trauma	Not dominant limb	77	44.0	24.8	*0.015*
	Dominant limb	73	52.1	17.7	
MCS post-trauma	Not dominant limb	77	57.6	14.0	**0.485**
	Dominant limb	73	57.9	7.6	

**Table 4:** Follow-up outcome measures stratified by comorbidities (Mann–Whitney U test)

	Group	*N*	Median	IQR	*P*-value
SSV	With comorbidities	105	80.0	25.0	**0.063**
	Without comorbidities	44	90.0	20.0	
OSS	With comorbidities	105	39.0	14.0	*0.015*
	Without comorbidities	44	46.0	10.0	
Quick dash	With comorbidities	105	15.0	33.0	*0.009*
	Without comorbidities	44	5.0	14.0	
PCS post-trauma	With comorbidities	105	44.3	23.8	*0.025*
	Without comorbidities	44	51.5	12.0	
MCS post-trauma	With comorbidities	105	57.1	12.6	**0.530**
	Without comorbidities	44	58.3	7.2	

Increasing patient age was correlated with poorer outcomes at both the time of injury and follow-up (Table 5). The strongest correlations were observed for OSS (r = -0.407 at injury, r = − 0.406 at follow-up; *p* < 0.001), QuickDASH (r = 0.399 at injury, r = 0.399 at follow-up; *p* < 0.001), and SF-12 PCS (r = − 0.504 at injury, r = − 0.460 at follow-up; *p* < 0.001). In contrast, follow-up duration was not significantly correlated with any functional outcome measures.

**Table 5:** Correlation between patient age and functional outcomes at the time of injury and follow-up (Spearman’s rank correlation coefficient)

		Age at injury	Age at follow-up
SSV	Spearman’s rho	− 0.304	− 0.299
	*p*-value	< *0.001*	< *0.001*
OSS	Spearman’s rho	− 0.407	− 0.406
	*p*-value	< *0.001*	< *0.001*
Quick dash	Spearman’s rho	0.399	0.399
	*p*-value	< *0.001*	< *0.001*
PCS pre-trauma	Spearman’s rho	− 0.504	− 0.460
	*p*-value	< *0.001*	< *0.001*
MCS pre-trauma	Spearman’s rho	− 0.197	− 0.228
	*p*-value	*0.016*	*0.005*

Pre-injury physical and mental health, assessed using the SF-12 PCS and MCS components, was significantly associated with post-trauma functional outcomes across all evaluated measures (Table 6).

**Table 6:** Correlation between pre-injury SF-12 PCS and MCS scores and functional outcomes (Spearman's rank correlation coefficient)

		PCS-12 pre-injury	MCS-12 pre-trauma
SSV	Spearman’s rho	0.473	0.367
	*p*-value	< *0.001*	< *0.001*
OSS	Spearman’s rho	0.647	0.412
	*p*-value	< *0.001*	< *0.001*
Quick dash	Spearman’s rho	− 0.594	− 0.354
	*p*-value	< *0.001*	< *0.001*
PCS post-trauma	Spearman’s rho	0.798	0.383
	*p*-value	< *0.001*	< *0.001*
MCS post-trauma	Spearman’s rho	0.282	0.822
	*p*-value	< *0.001*	< *0.001*

Radiographic characteristics were also associated with functional recovery. Patients with non-displaced (simple) fractures (64 fractures) had significantly better shoulder-specific outcomes and SF-12 PCS scores (all *p* < 0.001) compared to those with displaced fractures (86 fractures). In contrast, SF-12 MCS did not differ significantly between groups (*p* = 0.111) (Table 7). The number of fracture fragments (two, three or four) was not significantly associated with functional outcomes, regardless of fracture displacement status.

**Table 7:** Functional outcomes stratified by fracture composition. Mann–Whitney U test

	Group	*N*	Median	IQR	*P*-value
SSV	Displaced	86	80.0	27.5	< *0.001*
	Non-displaced	64	90.0	19.0	
OSS	Displaced	86	38.0	14.5	< *0.001*
	Non-displaced	64	46.0	9.5	
Quick dash	Displaced	86	15.6	33.5	< *0.001*
	Non-displaced	64	4.5	17.8	
PCS post-trauma	Displaced	86	39.6	23.1	< *0.001*
	Non-displaced	64	54.1	12.3	
MCS post-trauma	Displaced	86	56.8	12.7	**0.111**
	Non-displaced	64	59.3	5.0	

Within the subgroup of displaced fractures, correlation analyses confirmed the trends observed in the overall cohort. Higher pre-injury SF-12 scores, particularly the PCS component, were significantly associated with better post-trauma shoulder function (Table 8), with the strongest correlations observed for OSS (r = 0.655; *p* < 0.001) and post-trauma SF-12 PCS (r = 0.737; *p* < 0.001).

**Table 8:** Correlation between pre-injury SF-12 PCS and MCS scores and functional outcomes in patients with displaced fractures

		PCS-12 pre-trauma	MCS-12 pre-trauma
SSV	Spearman’s rho	0.487	0.346
	*p*-value	**< ** ***0.001***	< *0.001*
OSS	Spearman’s rho	0.655	0.466
	*p*-value	< *0.001*	< *0.001*
Quick Dash	Spearman’s rho	− 0.578	− 0.379
	*p*-value	< *0.001*	< *0.001*
PCS post-trauma	Spearman’s rho	0.737	0.391
	*p*-value	< *0.001*	< *0.001*
MCS post-trauma	Spearman’s rho	0.292	0.812
	*p*-value	*0.006*	< *0.001*

*N* = 86. Spearman’s rank correlation coefficient

Stratification according to limb dominance (Table 9) showed that displaced fractures involving the dominant arm consistently resulted in higher functional scores across all outcome measures. Conversely, displaced fractures of the non-dominant arm were associated with a greater decline in functional outcomes compared with the overall cohort. Patients with displaced fractures were older, with a mean age at the time of injury of 76.2 ± 7.9 years and a narrower age range (54–90 years) than the entire study population.

**Table 9:** Follow-up measurement outcomes in displaced fractures, stratified by dominant side (Mann–Whitney U test)

	Group	N	Mean	SD	p
SSV	Dominant limb	35	85.0	25.0	*0.024*
	Not dominant limb	51	75.0	40.0	
OSS	Dominant limb	35	42.5	12.5	*0.012*
	Not dominant limb	51	36.0	14.0	
Quick Dash	Dominant limb	35	9.7	21.7	*0.006*
	Not dominant limb	51	22.5	33.6	
PCS post-trauma	Dominant limb	35	51.5	23.3	*0.002*
	Not dominant limb	51	36.1	22.1	
MCS post-trauma	Dominant limb	35	55.9	9.0	**0.312**
	Not dominant limb	51	57.1	17.5	

Multivariable regression analysis (Table 10) identified gender, age at injury, and dominant limb involvement as independent predictors of OSS. Age at injury and dominant limb involvement were also independent predictors of SSV. In contrast, age, post-injury limb status, and fracture displacement were independently associated with PCS, whereas only age at injury and fracture displacement remained independently associated with QuickDASH.

**Table 10:** Multivariable analysis of independent predictors of functional outcomes after proximal humeral fractures

Predictor variable	SSV		OSS		Quick dash		PCS post-trauma	
	β (95% CI)	*p*	β (95% CI)	*p*	β (95% CI)	*p*	β (95% CI)	*p*
Gender (female vs male)	− 6.20 (− 14.11 to 1.74)	**0.123**	− 3,77 (− 6.62 to − 1.07)	*0.014*	5.37 (− 1.08 to 11.67)	**0.089**	− 0.60 (− 4.89 to 3.64)	**0.796**
Limb dominance (yes vs. no)	7.32 (1.60 to 13.55)	*0.016*	2.99 (0.63 to 6.09)	*0.048*	− 4.25 (− 9.56 to 0.82)	**0.159**	3.81 (0.95 to 7.57)	*0.046*
Comorbidities (yes vs no)	− 2.31 (− 9.29 to 5.34)	**0.538**	− 1.83 (− 4.99 to 1.53)	**0.262**	4.45(− 1.99 to 10.36)	**0.157**	− 2.75 (− 6.36 to − 0.95)	**0.148**
Fracture displacement (no vs yes)	4.19 (− 3.74 to 11.49)	**0.277**	2.43 (− 1.16 to 5.85)	**0.170**	− 9.23 (− 15.37 to − 3.14)	*0*.*003*	5.45 (1.66 to 9.09)	*0.008*
Age at injury (years)	− 3.82 (− 0.64 to − 0.14)	*0.007*	− 0.27 (− 0.40 to − 0.16)	< *0.001*	0.52 (0.26 to 0.81)	< *0.001*	− 0.36 (− 0.55 to − 0.18)	< *0.001*

### Surgeons’ recommendations

In the second phase of the study, experienced shoulder surgeons demonstrated high overall agreement in the assessment of fracture displacement. Among the 150 fractures evaluated, 62 were classified as non-displaced by the expert panel, compared with 64 fractures in the initial radiographic classification, resulting in a 93% concordance rate. Of the 88 fractures classified as displaced, a predominant recommendation for non-surgical treatment was made in 50 cases, while surgical treatment was recommended in the remaining 38 cases, with satisfactory concordance rates observed in both groups.

No statistically significant differences were observed in SSV, OSS, QuickDASH, or SF-12 (PCS and MCS) scores between patients for whom the expert surgeons predominantly recommended surgical treatment and those for whom conservative management was recommended (Table 11).

**Table 11:** Functional outcomes in patients with displaced fractures, stratified by treatment indication (Mann–Whitney U test)

	Group	N	Median	IQR	*P*-value
SSV	Surgery	38	75.0	30.0	**0.517**
	Conservative	50	82.5	21.3	
OSS	Surgery	38	38.0	12.0	**0.965**
	Conservative	50	38.0	17.3	
Quick dash	Surgery	38	15.0	32.9	**0.811**
	Conservative	50	16.7	34.6	
PCS post-trauma	Surgery	38	37.8	22.4	**0.225**
	Conservative	50	44.1	24.0	
MCS post-trauma	Surgery	38	56.8	12.5	**0.983**
	Conservative	50	56.9	15.3	

## Discussion

Proximal humerus fractures (PHFs) are among the most frequent injuries in the elderly population and represent a major cause of pain, disability and healthcare utilization [15–17]. Despite their high incidence and the extensive literature available, the optimal management of these fractures remains a subject of ongoing debate [16, 18].

The present study was conducted in two phases: the first evaluated long-term functional outcomes following non-surgical treatment of PHFs, while the second investigated the therapeutic decision-making of upper limb specialist orthopedic surgeons to determine whether retrospective treatment recommendations correlated with clinical outcomes in conservatively managed patients.

Our cohort consisted of 150 PHFs treated non surgically, all with a minimum follow-up of 12 months (range 22–49 months, mean 32.1 months). Multivariable regression analysis demonstrated that patient-related factors were more consistently associated with long-term outcomes than fracture-related characteristics. Increasing age remained independently associated with all patient-reported outcome measures, whereas female gender was independently associated with lower OSS scores. Dominant limb involvement was independently associated with better OSS and SSV scores. Among fracture-related variables, fracture displacement independently predicted poorer PCS and QuickDASH scores, while fracture fragmentation was not independently associated with any outcome measure.

The influence of age is consistent with previous evidence demonstrating that advancing age adversely affects recovery following PHFs owing to reduced bone quality, sarcopenia, diminished physiological reserve, and lower rehabilitation potential [14, 15, 18] Although bone quality was not directly assessed in the present study, the observed association aligns with the well-established epidemiology of PHFs, which occur predominantly in older women with skeletal fragility. Similarly, the independent association between female gender and lower OSS scores may reflect the higher prevalence of osteoporosis and frailty among elderly women rather than gender itself. Systematic reviews by Handoll and colleagues and prognostic studies by Beks et al. have likewise emphasized that age, frailty, and baseline health status are major determinants of recovery irrespective of treatment strategy [15, 16, 19]

Patients with comorbidities demonstrated significantly worse unadjusted functional outcomes, including lower OSS, QuickDASH, and SF-12 PCS scores. However, comorbidity did not remain independently associated with outcome after adjustment for other variables. This finding suggests that the effect of comorbidity may be largely mediated through age, physical function, or overall frailty rather than representing an independent prognostic factor. This interpretation is consistent with the EPIDOS prospective study, which highlighted global health status and functional reserve, rather than individual diseases, as key determinants of fracture-related outcomes [20]. Taken together, these results suggest that comorbidities may primarily act as markers of general frailty and reduced physiological reserve, rather than as isolated predictors of functional recovery after PHFs. Consequently, the impact of comorbidities on outcome appears to be mediated by the patient’s overall functional status and baseline quality of life, rather than by the effect of specific pathological conditions.

Among radiographic characteristics, fracture displacement emerged as the only fracture-specific variable independently associated with poorer functional recovery, predicting worse PCS and QuickDASH scores. In contrast, the number of fracture fragments was not independently associated with any outcome measure. These findings support previous observations by Beraldo et al., who identified fracture displacement as a more relevant prognostic factor than fracture complexity in elderly patients treated conservatively [21]. Collectively, these results suggest that displacement more accurately reflects the mechanical severity of injury than fragment count alone. However, interpreting this finding solely through a mechanical lens would overlook a fundamental selection bias: it is highly probable that more complex and displaced fracture patterns occur in patients who are inherently frailer. In such individuals, even low-energy trauma can result in severe displacement due to compromised bone quality and diminished physiological reserve. While a methodologically ideal comparison would involve evaluating morphologically identical fractures across populations with differing baseline health statuses, the multifactorial nature of general well-being involves too many variables to be quantified objectively without significant confounding.

An interesting finding of the present study was the independent association between dominant limb involvement and better OSS and SSV scores. Although the influence of limb dominance remains controversial, this observation may reflect greater patient motivation, higher compliance with rehabilitation, superior baseline function of the dominant upper extremity, or more effective functional adaptation during recovery rather than a direct biomechanical effect. Previous studies have reported that limb dominance does not necessarily affect functional outcomes or quality of life after PHFs, even when non surgically managed, indicating that dominance alone may not be a strong independent predictor of prognosis [22]. However, other evidence has highlighted the relevance of functional factors and habitual use of the dominant limb in the recovery process, suggesting that patients may achieve better rehabilitation performance and functional adaptation when the dominant arm is involved [15, 23]. In this context, the present results may reflect the interaction between limb dominance and patient-related factors, such as baseline functional status, motivation, and adherence to rehabilitation, rather than a direct biomechanical effect of dominance itself. These findings support the hypothesis that limb dominance may play a contributory, but not decisive, role in functional recovery after non-surgical treatment of proximal humerus fractures and should be interpreted in combination with other clinical and radiographic determinants of outcome.

These findings provide important context for interpreting the second phase of the study, which evaluated the retrospective treatment recommendations of shoulder specialists. Although displaced fractures were more frequently considered surgical candidates, functional outcomes among patients with displaced fractures did not differ significantly between those whom specialists retrospectively judged appropriate for surgery and those for whom conservative management would have been maintained (Table 11). This observation suggests that, once a threshold of morphological severity has been reached, long-term outcome may depend more on patient-related biological and functional factors than on the theoretical indication for surgery.

In the present study, only shoulder specialists participated in the decision-making process. This methodological choice is relevant because previous studies have shown that treatment recommendations for PHFs vary according to surgeons’ training and sub-specialization. Shoulder surgeons and trauma surgeons often diverge in their therapeutic approach, with shoulder surgeons generally favoring surgical solutions in selected fracture patterns and patient profiles [25, 26].

These findings align with and extend the results of the ProFHER trial, which demonstrated no functional superiority of surgery over conservative treatment for displaced PHFs while reporting higher complication rates among surgically treated patients [17]. Similarly, the Cochrane reviews by Handoll et al. [15, 16] concluded that conservative treatment provides outcomes comparable to surgery for many displaced fractures, particularly in elderly patients, and emphasized the importance of patient-related characteristics over fracture morphology alone. More recent systematic reviews and meta-analyses have reached similar conclusions, reporting no clinically meaningful functional advantage of surgery while consistently identifying higher complication rates after operative treatment [18, 24]. Furthermore, evidence suggests that rehabilitation strategies, including early mobilization, may have a greater influence on recovery than the choice between operative and non-operative treatment in appropriately selected patients [24].

Overall, the present study reinforces the growing body of evidence supporting individualized treatment strategies for PHFs Although fracture displacement remains an important consideration, patient age, biological reserve, and functional characteristics appear to be more consistent determinants of recovery after conservative treatment [27]. These findings support a patient-centered approach to treatment selection and suggest that surgical decision-making should integrate patient-related prognostic factors alongside fracture morphology rather than relying predominantly on radiographic appearance.

### Limitations

This study has several limitations. First, only conservatively treated PHFs were included, unlike large comparative studies such as ProFHER, which analyzed both surgically and non-surgically treated fractures. Consequently, our findings are applicable only to patients treated nonoperatively, limiting their external validity and generalizability.

Second, more than half of the initially eligible patients were lost to follow-up, which may have introduced selection bias. In addition, no formal a priori sample size calculation was performed. The final cohort comprised 150 patients and may have been underpowered to detect small differences in outcomes related to subgroups and surgeon clinical judgment. Accordingly, the analyses should be considered exploratory and hypothesis-generating.

Third, pre-injury quality of life was assessed retrospectively, requiring patients to recall their pre-injury health status at the time of follow-up, in the absence of prospectively collected baseline data, thereby introducing the potentisl for recall bias. Additionally, because of the retrospective study design, CT scans were not available for all patients during the second phase of the study.

Taken together, these limitations reflect the observational and explorative nature of the study and the challenges inherent in long-term research. Nevertheless, the findings provide clinically relevant insights into factors associated with long-term functional recovery and may help inform decision-making for patients with non-surgically treated PHFs.

## Conclusions

In PHFS treated conservatively, patient-related characteristics exerted a greater influence on long-term functional recovery than fracture morphology alone. While fracture displacement independently affected physical function and disability, age remained the only variable consistently associated with all outcome measures, highlighting the central role of biological and functional reserve in determining recovery after non-surgical management of PHFs.

Even in displaced fractures, non-surgical treatment can provide satisfactory results in most cases. The experience of the orthopedic shoulder specialists, when retrospectively applied to treatment decision-making, appears to have limited prognostic value.

These findings support a patient-centered approach emphasizing functional demands, general health, and rehabilitation potential over fracture complexity alone.

## Data Availability

No datasets were generated or analysed during the current study.

## Abbreviations

PHFs: Proximal humerus fractures
QuickDASH: Quick disabilities of the arm, shoulder and hand
OSS: Oxford shoulder score
SSV: Subjective shoulder value
SF-12: Short form-12 health survey
PCS: Physical component score
MCS: Mental component score
